# Immunisation: A Criticism of Current Theory and Practice

**Published:** 1909-07-10

**Authors:** 


### SPECIAL ARTICLE.

### IMMUNISATION: A CRITICISM OF CURRENT THEORY AND PRACTICE.

(From a Correspondent.)

The disappointing results of vaccine treatment in tuberculosis and other infections would seem to necessitate a reconsideration of the claims put forward on its behalf, and of the mode in which its activities have been supposed to be exercised.

A volume recently published from the pen of Dr. E. C. Hort, entitled "Rational Immunisation in Tuberculosis," contains valuable criticisms on the subject, and views on immunisation, derived from personal investigations, which are worthy of the most serious attention. Dr. Hort does not hesitate to maintain that the current vaccine treatment of all kinds of infections, except relatively unimportant ones, is a failure, and that such failure is inevitable in all serious affections accompanied by extensive tissue changes.

Up to the present the vaccinists and opsonists appear to have assumed that a specific disease is due to its specific organism alone, and that cellular antagonism may be practically ignored. Yet it is fairly obvious, at least to the present writer, that bacterial vaccines can induce bacterio-tropic response, and nothing more.

Vaccine therapy entirely ignores the necessity for exciting cellulo-tropic response. This is surely a limited view of the production of immunity. A wider conception is that, in order to create or restore immunity to the body as a whole, whatever the infection, the reaction of the tissue cells to intrinsic stimuli must be aroused, as well as to the extrinsic ones exerted by bacteria and toxins. These intrinsic stimuli to cell-reaction include, among others, the intra-cellular enzymes, some of which are autolytic, and the products of their morbid activity.

Under certain circumstances these enzymes and their products are highly toxic and even autotoxic. Such an admission leads to a generalisation of enormous consequence. Usually it is assumed that the toxæmia of infective disease proceeds only from the invading organism. This cannot be admitted as the only cause, for there is evidence to show that the toxic symptoms may proceed as much from the tissue cell derivatives as from the bacterial ones. Indeed, it is more than possible that cellular changes often precede infection, and thus explain the, at present, inexplicable conversion of non-pathogenic organisms into an active state of pathogenicity. The pneumo-coccus, the organisms of infectious coryza, and the colon bacillus are common instances of this change. The wide distribution of the tubercle bacillus and the frequency with which healed tuberculous lesions are found after death bring this organism into the same category.

It is probable that few individuals escape infection at some period of life, but by making use of a process of spontaneous auto-inoculation recovery ensues. The cellulo-tropic restraint is sufficient protection in the vast majority of infections. Measures for the prevention of tuberculosis must be directed to raising this resistance, as well as reducing the chances of infection. Tuberculin administration, whether by mouth or subcutaneously, is necessarily of very limited value, and is frequently most disappointing. It makes no provision for immunising the lung against itself.

Most practitioners have found instances in which tuberculin administration is ignored absolutely by the economy, whether for good or evil, even in cases where the disease is active. This is not surprising, for no amount of tuberculo-opsonin or other purely antibacterial substance can enable already damaged cells to recover.

It is the firm conviction of the present writer that if the cells are not irretrievably damaged, recovery can be effected by inciting intracellular restraint, and by this method alone. No doubt immunising the cells against the bacillus and its toxins may prevent further infection and progressive damage, but it does not affect the damage already done. Even if it is assumed that the tubercle bacillus is entirely responsible for the tissue changes of tuberculosis, anti-tuberculous bodies can only act indirectly in enabling the infected lungs to recover from the infection.

Though in certain infections, notably mild staphylococcal ones of the skin, bacterial vaccine therapy has a definite value, there is much too great a tendency to expect more from the method than is reasonably possible.

If vaccines were made from cellular emulsions, it seems at least possible that the present fatal objections raised against bacterial injections on a large scale would be met, on the assumption that such emulsions would undoubtedly have a cellulo-tropic restraint.

Such a procedure is unnecessary, for much better results have been obtained by the methods of auto-inoculation, the infected cells and the infecting organisms being used as natural vaccines, thus exciting a double response. This method has been carried out with great success in apyrexial tuberculosis by Paterson's method of graduated labour; and by Hort in pyrexial cases, by induction of artificial hyperæmia of the lungs.

Nature cures infection by converting both cells and bacteria into auto-inoculating agents, and thus exerting both cellular and bacterial restraint. To confine attention to the bacterial and ignore the cellular aspect leads to failure, or at best to a most partial success in treatment.

These considerations deserve the most serious attention of vaccinists, opsonists, and medical practitioners. It is perhaps not too much for the present author to expect that they will lead to the vaccine therapeutists becoming a little more modest in their views.

